# Automatic and Efficient Prediction of Hematoma Expansion in Patients with Hypertensive Intracerebral Hemorrhage Using Deep Learning Based on CT Images

**DOI:** 10.3390/jpm12050779

**Published:** 2022-05-12

**Authors:** Chao Ma, Liyang Wang, Chuntian Gao, Dongkang Liu, Kaiyuan Yang, Zhe Meng, Shikai Liang, Yupeng Zhang, Guihuai Wang

**Affiliations:** 1 School of Clinical Medicine, Tsinghua University, Beijing 100084, China; mac21@mails.tsinghua.edu.cn (C.M.); wly21@mails.tsinghua.edu.cn (L.W.); gct21@mails.tsinghua.edu.cn (C.G.); yky19@mails.tsinghua.edu.cn (K.Y.); mengz20@mails.tsinghua.edu.cn (Z.M.); 2 Department of Neurosurgery, Beijing Tsinghua Changgung Hospital, School of Clinical Medicine, Tsinghua University, Beijing 102218, China; ldka02487@btch.edu.cn (D.L.); lska02140@btch.edu.cn (S.L.); 3 Interventional Neuroradiology Center, Beijing Tiantan Hospital, Capital Medical University, Beijing 100050, China; 4 Beijing Neurosurgical Institute, Capital Medical University, Beijing 100050, China

**Keywords:** hematoma expansion, hypertension, end-to-end, deep learning

## Abstract

Patients with hypertensive intracerebral hemorrhage (ICH) have a high hematoma expansion (HE) incidence. Noninvasive prediction HE helps doctors take effective measures to prevent accidents. This study retrospectively analyzed 253 cases of hypertensive intraparenchymal hematoma. Baseline non-contrast-enhanced CT scans (NECTs) were collected at admission and compared with subsequent CTs to determine the presence of HE. An end-to-end deep learning method based on CT was proposed to automatically segment the hematoma region, region of interest (ROI) feature extraction, and HE prediction. A variety of algorithms were employed for comparison. U-Net with attention performs best in the task of segmenting hematomas, with the mean Intersection overUnion (mIoU) of 0.9025. ResNet-34 achieves the most robust generalization capability in HE prediction, with an area under the receiver operating characteristic curve (AUC) of 0.9267, an accuracy of 0.8827, and an F_1_ score of 0.8644. The proposed method is superior to other mainstream models, which will facilitate accurate, efficient, and automated HE prediction.

## 1. Introduction

Studies showed that the incidence of ICH today is as high as 3 in 10,000, which already accounts for 10–15% of all strokes [1,2,3]. In patients with hypertension, cerebral blood vessels rupture due to a sudden increase in blood pressure, such as intracranial hemorrhage and subarachnoid hemorrhage. Patients present with severe headache, dizziness, nausea, projectile vomiting, and sudden severe loss of consciousness [4,5]. Notably, HE is a serious and typical complication of hypertensive ICH, with an incidence of approximately one-third [6,7,8]. HE also has an important impact on the prognosis of the patient’s nervous system. Therefore, early detection of high-risk patients can help doctors to take necessary measures in a timely manner, such as controlling hypertension, reducing intracranial pressure, and administering hemostatic and coagulation drugs so as to prevent accidents [9,10].

How to non-invasively predict HE has become a hot spot in clinical research. Traditionally, hypertension, CT scan images, and warfarin use [11,12] are independent clinical factors predicting HE. Additionally, radiological indicators such as CT angiography point sign [13,14], leakage sign [14], island sign [15], mixed sign [16,17], black hole sign [18], and whirlpool sign [19] are also helpful for doctors to detect HE. However, the above qualitative criteria can only be roughly estimated and cannot be accurately predicted for individuals, which confuses doctors in their decision-making.

With the development of artificial intelligence (AI) technology, scientists have combined it with medical imaging to identify diseases better. CT images of patients with ICH show that enlarged hematomas are more prone to heterogeneity in shape, texture, and signal intensity. Based on this, patients’ CT images and corresponding clinical data were collected to develop automatic AI models for the accurate prediction of HE.

This paper proposed a deep learning-based end-to-end method for predicting HE. For CT data, we employed the U-net algorithm with attention to automatically segment the hematoma area after preprocessing. After that, the convolutional neural networks (CNNs) were applied to predict HE. By comparison, it is found that ResNet-34 performs the best in the test set, which indicates that the algorithm has excellent generalization performance in predicting HE. This is the first report using deep learning for end-to-end prediction of HE (integrating automatic segmentation and classification) to the best of our knowledge.

## 2. Materials and Methods

**Figure 1:**
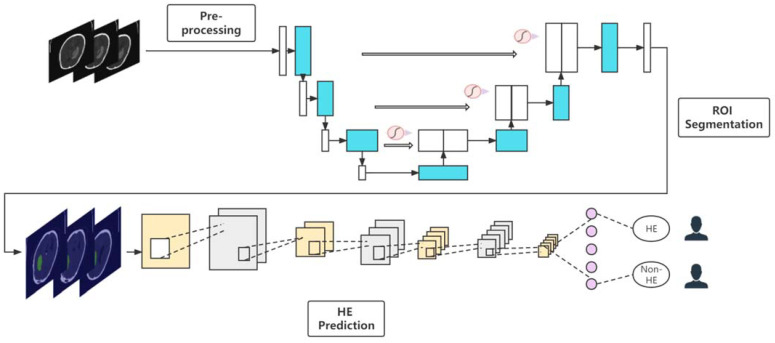
The workflow of this end-to-end deep learning method.

The workflow for this study is shown in Figure 1.

### 2.1. Patient’s Demographics and Data Acquisition

The Ethics Committee of Beijing Tiantan Hospital approved this study (ethical statement number: KY2020-112-02). All participants obtained written informed consent. Patients with ICH who had baseline NECT scans within 6 h of onset of symptoms and follow-up scans within 48 h of baseline scans from January 2015 to July 2018 were retrospectively analyzed. The following patient exclusion criteria were set: secondary ICH (arteriovenous malformation, aneurysm, head trauma, brain tumor); surgical treatment prior to follow-up scan; hemorrhagic evolution of ischemic infarction; primary intraventricular hemorrhage; artifacts present; cerebellum/brain stem hematoma; and anticoagulant/antiplatelet therapy was administered. In this study, HE was defined as an absolute increase of more than 12.5 mL or a relative increase of more than 33% in the repeat results compared to the original volume [7].

### 2.2. CT Examination and Image Analysis

Baseline NECT images were collected by GE Healthcare within 6 h of symptom onset. Follow-up NECTs were then obtained again with the same device within 48 h. The scan energy of the device was 120 KVP, and smart mAs was selected. All slices were 5.0 mm thick with a pixel pitch of 0.45 × 0.45 mm^2^.

CT scans ranged from −1000 to 400HU (normalized operation) in this work. Some slices without ROI in the original CT increase the computational complexity and be ineffective for model training. We cropped out unmarked slices based on manually annotated data (described below). The input channels of the segmentation and classification models used in this paper are all two-dimensional, so the data are also converted into the corresponding format. In addition, data augmentation operations, such as horizontal flipping, random rotation, random blurring, etc., were performed on the divided training set before executing the task, which can avoid the performance degradation of the model caused by the uneven number of different types.

### 2.3. Manual Annotation

The segmentation of this study employed supervised learning, so four radiologists with ten years or more of experience completed the manual labeling task. Among them, three doctors delineated the bleeding location of each sample with the help of 3D Slicer (Boston, MA, USA) software, and the other doctor reviewed the results of the former marking.

### 2.4. Segmentation Models Construction

In this study, supervised learning was employed to automatically segment the hematoma region. After analyzing the image features, the U-Net deep learning architecture was chosen. U-Net, U-Net++, and U-Net with attention were proposed for training and testing.

#### 2.4.1. U-Net with Attention

Attention U-Net was published in 2018 [20]; it was verified to perform well in several medical image segmentation tasks [21,22], but it has not yet been employed in brain hematoma segmentation.

This model proposed an Attention Gate (AG) structure, which is connected at the end of each skip connection to implement the attention mechanism for the extracted features. Based on the basic U-Net, the attention mechanism was added, and the activation value was adjusted by automatically learning parameters. The visualization effect of attention is still the main part, and unlike the non-local method, each pixel must be compared with other pixels. Therefore, it can be regarded as an implicit attention mechanism.

Attention U-net was set to two-dimensional input channels. The image size was converted to 512 × 512 in this work; meanwhile, the initial model was pre-train to directly adapt to the downstream hematoma region segmentation.

#### 2.4.2. U-Net++

U-Net++ is a new segmentation structure based on nested dense skip connections to solve the accuracy problem of medical image segmentation [23]. U-Net++ network is also composed of an encoder and decoder. Unlike U-Net, U-Net++ consists of an encoder and decoder connected by a series of nested dense convolution blocks. The main idea of this architecture is to bridge the semantic gap between the feature maps of the encoder and decoder before fusion. In this paper, the dimensions and sizes of the input images are the same as those of the above model and were also pre-trained.

#### 2.4.3. U-Net

As a classic medical image segmentation model, U-Net has been widely used in various tasks [24,25]. The architecture is a classic fully convolutional network (i.e., no fully connected operations in the network). This study compared it with the aforementioned models, such as Attention U-Net and U-Net++.

### 2.5. Prediction Models Construction

To automatically extract the features of the hematoma area and achieve accurate classification, CNN models were applied. This study adopted two mainstream deep learning architectures, ResNet and Visual Geometry Group (VGG), which perform well in multiple medical image recognition tasks [26,27,28] but are less adopted in HE prediction.

A residual structure is proposed in ResNet, which effectively solves the degradation problem of traditional CNNs as the network becomes deeper. The residual structure employs a shortcut connection method; that is, the feature matrix is added at intervals. In addition, the proposal of batch normalization (BN) layer effectively avoids gradient vanishing or gradient explosion. In this experiment, ResNet-18 and ResNet-34 were respectively constructed and applied to the prediction of HE. Notably, the segmented slices (the size was set to 512 × 512) were input to the model for training, while in the validation and test sections, the results from all slices were aggregated to predict patient outcomes.

In VGG, 3 and 2 3 × 3 convolution kernels are used to replace AlexNet’s 7 × 7 and 5 × 5 convolution kernels, respectively. The primary purpose of this is to deepen the depth of the network under the condition of ensuring the same perceptual field, thereby improving the effectiveness of the neural network to a certain extent. For a given receptive field, employing stacked small convolution kernels outperforms large convolution kernels. Multiple non-linear layers can increase the depth of the network to ensure learning more complex patterns with relatively few parameters. VGG-16 was built as a prediction model for HE in the study. Likewise, we set the input size of the image to 512 × 512.

### 2.6. Statistics and Evaluation

For clinical indicators of patients, differences were calculated using a Student’s *t*-test or Mann–Whitney U-test. By considering the distribution of continuous data (normal distribution or not), the mean ± standard deviation or the median of the interquartile range was calculated as a result. Differences in categorical variables were estimated employing the chi-square test, and results were presented as a number of events followed by relative frequencies (%). The criterion of significant difference was set as *p* < 0.05.

For deep learning evaluation, mIoU, accuracy (Acc), Kappa, and Dice coefficients were selected as the computational metrics for the segmentation models. Meanwhile, the prediction models introduced Acc, recall, precision (Prec), F_1_ score, receiver operating characteristic (ROC) curve, and the corresponding AUC as evaluation criteria. Means and corresponding 95% confidence intervals (CIs) were calculated. This study’s segmentation and classification tasks were binary classifications, and the threshold was set to 0.5.

### 2.7. Experiments

It should be emphasized that slices of the same patients may have high similarity. Data leakage may occur if slices are randomly divided directly, resulting in artificial performance improvement. Therefore, patients were randomly divided into a training set, validation set, and test set in a ratio of 8:1:1 during the segmentation. When the training was complete, the performance evaluation of models on the test set was carried out. The classification task employed a 5-fold cross-validation method for patients; thus, the dataset was randomly divided into 5 parts, 4 of which were used for training, and the rest were used for validation, repeated 5 times. After dividing according to the above criteria, convert the raw data into slices to enter the models.

The study trained the CT slices but validated and evaluated them on a patient-specific basis, which is clinically significant. The specific method was to summarize the predicted probabilities of all slices for each patient and take their average as the probability of the patient. All experiments were carried out using Windows 10 operating system. Related computing devices were configured with AMD Ryzen 7 5800H CPU (16 GB memory) and 2 GPUs, including NVIDIA^®^GeForce RTX 3070 and NVIDIA^®^Tesla V100 GPU with 32 GB memory. Both were supported by CUDA acceleration. All work was conducted using Python 3.8 and the deep learning framework Paddle-Paddle.

## 3. Results

### 3.1. Patient’s Demographics and Clinical Characteristics

**Figure 2:**
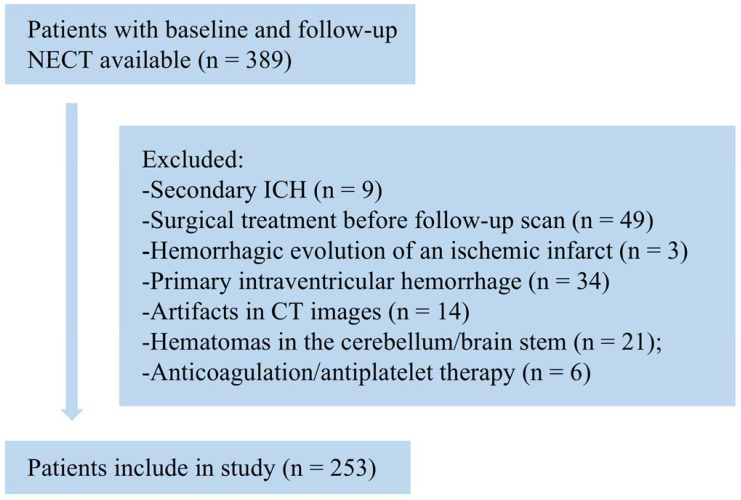
Criteria and statistics for the selection of retrospective data.

A preliminary collection of 389 patients with hypertensive intraparenchymal hematoma was used. The patient selection flowchart is shown in Figure 2. Nine patients had secondary ICH originating from arteriovenous malformations, aneurysms, head trauma, or brain tumors, and three patients with hemorrhagic evolution due to ischemic infarction were excluded. Forty-nine patients with the presence of surgical treatment prior to follow-up were also excluded. Thirty-four patients had predominantly intraventricular hematomas, and twenty-one patients who had hematomas located in the cerebellum and brainstem were excluded. Six and fourteen patients, respectively, who received anticoagulation/antiplatelet therapy and had CT images with artifacts that interfered with image interpretation were excluded.

A total of 253 patients were finally enrolled, including 157 males and 96 females, with an average age of 58.3 ± 13.0 years (age range 26–91 years), of which 57 (22.5%) patients had hematoma expansion. The HE group included 39 men and 18 women (median age, 57.0; IQR, 48.0–64.0), and the NHE group included 118 men and 78 women (median age, 59.0; IQR, 50.8–68.0). There were no statistically significant differences between the HE and NHE groups in terms of age (*p* = 0.179) and sex (*p* = 0.261). As for hematoma characteristics, hematoma volume (17.8 mL vs. 27.5 mL, *p* < 0.001), 3D diameter (47.5 mm vs. 57.5 mm, *p* < 0.001), and hematoma 2D diameter (40.6 mm vs. 49.8 mm, *p* < 0.001) were significantly smaller in the HE group. The clinical characteristics of the above patients are shown in Table 1.

### 3.2. Segmentation Results

These experiments employed supervised learning to train the training set and compare the final results. U-Net with attention, U-Net++, and U-Net were all iterated until the loss became stable and there was no overfitting. Momentum was selected as the optimizer and set to 0.9. Other key parameters such as initial learning rate, weight_decay, and batch size were set to 0.0001, 0.0003, and 2, respectively.

**Figure 3:**
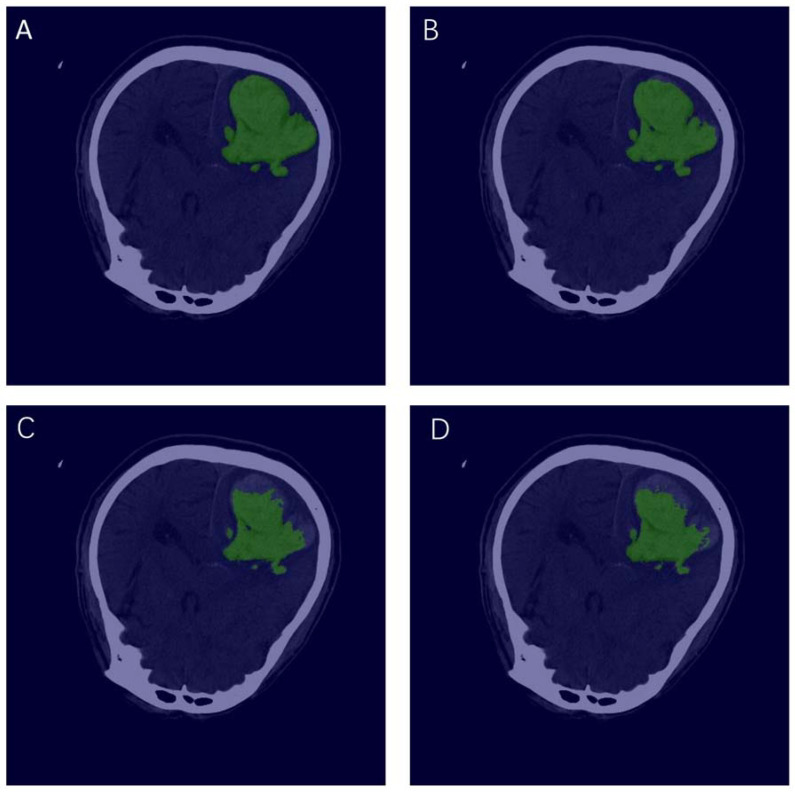
Visualization of manual labeling and each segmentation model. where (**A**) represents a manually labeled slice; (**B**–**D**) represent the result of the automatic segmentation of U-Net with attention, U-Net++, and U-Net, respectively.

Using the well-trained models for testing, Figure 3 shows the visualization of manual annotation and segmentation of each model. The green area represents the ROI. The evaluation indicators of the three segmentation models are shown in Table 2. It is not difficult to find that U-Net with attention has the best performance, with mIoU of 0.9025, Acc of 0.9976, kappa of 0.8922, and Dice of 0.9461. U-Net and U-Net++ perform similarly, where their mIoUs are 0.8847 and 0.8773, respectively.

It is worth noting that U-Net with attention also has the largest number of parameters, which increases the training difficulty.

### 3.3. Prediction Results

ResNet-18, ResNet-34, and VGG-16 deep learning models were adopted to automatically extract the texture features of the hematoma area and predict HE. The training was carried out according to the dataset mentioned above division ratio.

During training, key hyperparameters have been optimized (some parameters were set the same for the purposes of comparison). Among them, Adam was employed as the optimizer, which utilizes the first- and second-moment estimates of the gradient to dynamically adjust the learning rate of each parameter. The main advantage of Adam is that after bias correction, the learning rate has a certain range after each iteration, which makes the parameters relatively stable. The initial learning rate was set to 1.0 × 10^−5^, weight_decay was 0.001, verbose was 1, and batch size was set to 64.

After 10,000 iterations, all models converged without overfitting (the loss value tended to be stable and less than 0.001). The validation results at this time were selected, and the experiment was repeated five times to calculate the average value and the corresponding 95% CI of each model. The validation results of each model are shown in Table 3. It is not difficult to find that ResNet-34 performs the best among the three, with accuracy reaching 0.8827, higher than ResNet-18’s 0.8432 and VGG-16’s 0.8043. The F_1_ score is an important indicator used to measure the performance of the two-class model. It considers the accuracy and recall of the model at the same time. It can be regarded as a weighted average of the accuracy and recall, which is widely representative. ResNet-34 has the highest F_1_ score (0.8644) in this study, indicating that the model has the most robust generalization ability in predicting HE.

**Figure 4:**
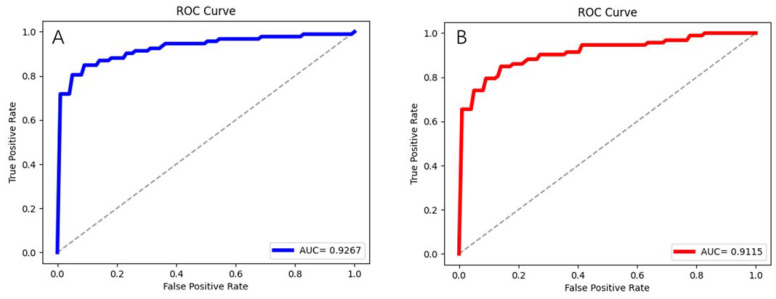

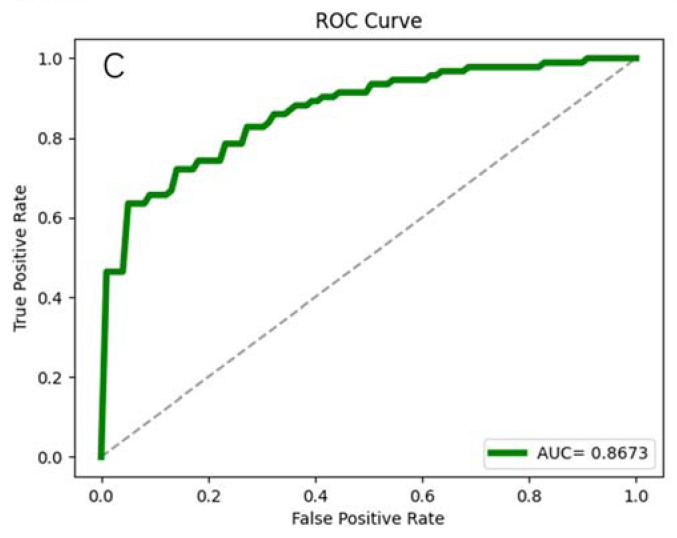
ROC and corresponding AUC of each prediction model on the validation set. where (**A**–**C**) represent the results of ResNet-34, ResNet-18, VGG-16, respectively.

The average ROC curves and their AUC values are shown in Figure 4 are another important indicator reflecting the performance of the models. The AUC value of ResNet-34 is still the highest, reaching 0.9267, followed by ResNet-18 (0.9115) and VGG-16 (0.8673).

## 4. Discussion

This paper constructed deep learning models to accurately predict whether HE occurs in patients with cerebral hemorrhage with hypertension. The deep learning framework set up automatic segmentation, extracted imaging features, and classified them. Through comparison, it was found that the best performance in the segmentation models is U-Net with attention, reaching 0.9025 mIoU. ResNet-34 performs best in predicting HE, with an AUC of 0.9267. This is the first report to integrate preprocessing, automatic segmentation, feature extraction, and HE prediction.

Attention U-Net introduced an attention mechanism based on U-Net, using an attention module to readjust the output features of the encoder before stitching the features on each resolution of the encoder with the corresponding features in the decoder. The module generates a gated signal that controls the importance of features at different spatial locations. This mechanism focuses attention on the target region, which is simply to make the value of the target region larger. U-Net with attention performs well in the segmentation tasks of medical imaging, which is closely related to its model architecture. This study introduced it into the HE classification and found that this model is superior to traditional U-Net and U-Net++.

ResNet-34 outperforms ResNet-18 and VGG-16 in this experiment. We believe that there are the following reasons. First, the input sample size reaches 1985 slices, and the texture characteristics of the hematoma area are more complex, which makes the training more difficult. As a result, ResNet-34 is more able to capture depth texture features because of the complexity of its model. Secondly, the residual structure solves the problem of network degradation, which is conducive to improving the prediction ability of HE.

By considering the small amount of data and the difficulty of training, this study built models for slices without setting the 3D input channel. A total of 253 patients were included in the study, and if directly targeted at patients, there may be an inability to converge due to a sample size that is too small. In addition, the input of 3D significantly increases the difficulty of training, which puts forward higher requirements for computing equipment. Based on this, this work proposed a method for training slices and predicting individual patients.

There are also reports of employing machine learning to predict HE based on radiomics. Liu et al. [29] retrospectively collected 1157 patients with spontaneous ICH. All hematoma areas were manually segmented. The study developed an SVM machine learning model to predict HE, where the AUC is 0.89. Cheng et al. [30] adopted a bimodal machine learning strategy to predict HE and obtained the highest AUC of 0.73. The method is similar to this paper, in which 5616 segmented hematoma images (from 104 patients) were trained, and individuals were predicted after summarizing the slices of each patient. Multiple logistic regression (LR) was employed to construct clinical radiology models for predicting HE tasks [31]. The study included 261 patients, eventually reaching an AUC of 0.867 in the validation cohort. A deep learning method was developed and validated to predict HE in patients with intracerebral hemorrhage [32]. The investigators retrospectively analyzed 1899 non-contrast computed tomography (NCCT) images of 118 patients with intracerebral hemorrhage and established a prediction model. The average AUC of this model reaches 0.780. Our work included only 253 patients, while the end-to-end deep learning method developed reached a maximum AUC of 0.9267.

There is no denying that this work is also flawed. For example, prediction based on 2D data cannot capture spatial information between slice and slice, which can affect the accuracy of decision-making. Second, the inputs in this study only considered brain CTs of patients and did not include other clinical indicators, which may also reduce the model’s accuracy. Furthermore, deficiencies such as the small sample size from the one center need to be continuously improved in the future. Additionally, the proposed method has not yet been used in clinical practice, which requires further attempts in the future.

## 5. Conclusions

Aiming at the high risk of HE occurrence in ICH patients, this study proposed an end-to-end deep learning method for automatic segmentation of hematomas, deep feature extraction, and HE prediction. It was found that U-Net with attention performs best in the segmentation task, reaching a mIoU of 0.9025. ResNet-34 is the most accurate predictor of HE, with an AUC of 0.9267. In the future, we will further expand the dataset to verify the applicability of the method and develop related software to serve radiologists.

## Figures and Tables

**Table 1:** Statistical results of clinical indicators of ICH patients.

Characteristics	HE Group	NHE Group	*p*
Patients, No. (%)	57 (22.5%)	196 (74.5%)	-
Age, y, median, (IQR)	57.0 (48.0–64.0)	59.0 (50.8–68.0)	0.179
Sex, M/F	39/18	118/78	0.261
Hematoma Volume (mL), mean, SD	17.8 (16.3)	27.5 (21.7)	<0.001
Hematoma Maximum 3D shape diameter (mm), mean, SD	47.5 (15.4)	57.5 (17.3)	<0.001
Hematoma Maximum 2D slice diameter (mm), mean, SD	40.6 (15.2)	49.8 (16.9)	<0.001

Note: SD represents standard deviation; IQR represents interquartile range.

**Table 2:** Comparison of model segmentation results.

Model	mIoU	Acc	Kappa	Dice	Parameter
U-Net attention	0.9025	0.9976	0.8922	0.9461	34,894,262
U-Net++	0.8773	0.9969	0.8605	0.9303	8,368,872
U-Net	0.8847	0.9971	0.8700	0.9350	13,404,354

**Table 3:** The performance of different prediction models on the validation set.

Model	Acc	Recall	Prec	F_1_ Score
ResNet-34	0.8827 ± 0.0562	0.8281 ± 0.0703	0.9058 ± 0.0699	0.8644 ± 0.0663
ResNet-18	0.8432 ± 0.0216	0.7304 ± 0.0344	0.9063 ± 0.0293	0.8086 ± 0.0306
VGG-16	0.8043 ± 0.0441	0.7199 ± 0.1316	0.8431 ± 0.0629	0.7629 ± 0.0713

Note: Acc and Prec are abbreviations for accuracy and precision, respectively.

## Data Availability

Not applicable.

## References

[1] Qureshi A.I., Tuhrim S., Broderick J.P., Batjer H.H., Hondo H., Hanley D.F. (2001). Spontaneous intracerebral hemorrhage. N. Engl. J. Med..

[2] Sudlow C.L., Warlow C.P. (1997). Comparable studies of the incidence of stroke and its pathological types: Results from an international collaboration. International Stroke Incidence Collaboration. Stroke.

[3] Feigin V.L., Lawes C.M., Bennett D.A., Anderson C.S. (2003). Stroke epidemiology: A review of population-based studies of incidence, prevalence, and case-fatality in the late 20th century. Lancet Neurol..

[4] Hansen B.M., Nilsson O.G., Anderson H., Norrving B., Saveland H., Lindgren A. (2013). Long term (13 years) prognosis after primary intracerebral haemorrhage: A prospective population based study of long term mortality, prognostic factors and causes of death. J. Neurol. Neurosurg. Psychiatry.

[5] Poon M.T., Fonville A.F., Al-Shahi Salman R. (2014). Long-term prognosis after intracerebral haemorrhage: Systematic review and meta-analysis. J. Neurol. Neurosurg. Psychiatry.

[6] Davis S.M., Broderick J., Hennerici M., Brun N.C., Diringer M.N., Mayer S.A., Begtrup K., Steiner T., Recombinant Activated Factor VIIIHTI (2006). Hematoma growth is a determinant of mortality and poor outcome after intracerebral hemorrhage. Neurology.

[7] Delcourt C., Huang Y., Arima H., Chalmers J., Davis S.M., Heeley E.L., Wang J., Parsons M.W., Liu G., Anderson C.S. (2012). Hematoma growth and outcomes in intracerebral hemorrhage: The INTERACT1 study. Neurology.

[8] Fujii Y., Tanaka R., Takeuchi S., Koike T., Minakawa T., Sasaki O. (1994). Hematoma enlargement in spontaneous intracerebral hemorrhage. J. Neurosurg..

[9] Anderson C.S., Heeley E., Huang Y., Wang J., Stapf C., Delcourt C., Lindley R., Robinson T., Lavados P., Neal B. (2013). Rapid blood-pressure lowering in patients with acute intracerebral hemorrhage. N. Engl. J. Med..

[10] Qureshi A.I., Palesch Y.Y., Barsan W.G., Hanley D.F., Hsu C.Y., Martin R.L., Moy C.S., Silbergleit R., Steiner T., Suarez J.I. (2016). Intensive Blood-Pressure Lowering in Patients with Acute Cerebral Hemorrhage. N. Engl. J. Med..

[11] Balami J.S., Buchan A.M. (2012). Complications of intracerebral haemorrhage. Lancet Neurol..

[12] Brouwers H.B., Chang Y., Falcone G.J., Cai X., Ayres A.M., Battey T.W., Vashkevich A., McNamara K.A., Valant V., Schwab K. (2014). Predicting hematoma expansion after primary intracerebral hemorrhage. JAMA Neurol..

[13] Demchuk A.M., Dowlatshahi D., Rodriguez-Luna D., Molina C.A., Blas Y.S., Dzialowski I., Kobayashi A., Boulanger J.M., Lum C., Gubitz G. (2012). Prediction of haematoma growth and outcome in patients with intracerebral haemorrhage using the CT-angiography spot sign (PREDICT): A prospective observational study. Lancet Neurol..

[14] Orito K., Hirohata M., Nakamura Y., Takeshige N., Aoki T., Hattori G., Sakata K., Abe T., Uchiyama Y., Sakamoto T. (2016). Leakage Sign for Primary Intracerebral Hemorrhage: A Novel Predictor of Hematoma Growth. Stroke.

[15] Li Q., Liu Q.J., Yang W.S., Wang X.C., Zhao L.B., Xiong X., Li R., Cao D., Zhu D., Wei X. (2017). Island Sign: An Imaging Predictor for Early Hematoma Expansion and Poor Outcome in Patients with Intracerebral Hemorrhage. Stroke.

[16] Li Q., Zhang G., Huang Y.J., Dong M.X., Lv F.J., Wei X., Chen J.J., Zhang L.J., Qin X.Y., Xie P. (2015). Blend Sign on Computed Tomography: Novel and Reliable Predictor for Early Hematoma Growth in Patients with Intracerebral Hemorrhage. Stroke.

[17] Sporns P.B., Schwake M., Schmidt R., Kemmling A., Minnerup J., Schwindt W., Cnyrim C., Zoubi T., Heindel W., Niederstadt T. (2017). Computed Tomographic Blend Sign Is Associated with Computed Tomographic Angiography Spot Sign and Predicts Secondary Neurological Deterioration after Intracerebral Hemorrhage. Stroke.

[18] Li Q., Zhang G., Xiong X., Wang X.C., Yang W.S., Li K.W., Wei X., Xie P. (2016). Black Hole Sign: Novel Imaging Marker That Predicts Hematoma Growth in Patients with Intracerebral Hemorrhage. Stroke.

[19] Ng D., Churilov L., Mitchell P., Dowling R., Yan B. (2018). The CT Swirl Sign Is Associated with Hematoma Expansion in Intracerebral Hemorrhage. AJNR Am. J. Neuroradiol..

[20] Vahadane A., Atheeth B., Majumdar S. (2021). Dual Encoder Attention U-net for Nuclei Segmentation. Annu. Int. Conf. IEEE Eng. Med. Biol. Soc..

[21] Hui H., Zhang X., Wu Z., Li F. (2021). Dual-Path Attention Compensation U-Net for Stroke Lesion Segmentation. Comput. Intell. Neurosci..

[22] Lin H., Li Z., Yang Z., Wang Y. (2021). Variance-aware attention U-Net for multi-organ segmentation. Med. Phys..

[23] Jin J., Zhu H., Zhang J., Ai Y., Zhang J., Teng Y., Xie C., Jin X. (2021). Multiple U-Net-Based Automatic Segmentations and Radiomics Feature Stability on Ultrasound Images for Patients with Ovarian Cancer. Front. Oncol..

[24] Falk T., Mai D., Bensch R., Çiçek Ö., Abdulkadir A., Marrakchi Y., Böhm A., Deubner J., Jäckel Z., Seiwald K. (2019). U-Net: Deep learning for cell counting, detection, and morphometry. Nat. Methods.

[25] Su R., Zhang D., Liu J., Cheng C. (2021). MSU-Net: Multi-Scale U-Net for 2D Medical Image Segmentation. Front. Genet..

[26] Sitaula C., Hossain M.B. (2021). Attention-based VGG-16 model for COVID-19 chest X-ray image classification. Appl. Intell..

[27] Yu X., Kang C., Guttery D.S., Kadry S., Chen Y., Zhang Y.D. (2021). ResNet-SCDA-50 for Breast Abnormality Classification. IEEE/ACM Trans. Comput. Biol. Bioinform..

[28] Yaqoob M.K., Ali S.F., Bilal M., Hanif M.S., Al-Saggaf U.M. (2021). ResNet Based Deep Features and Random Forest Classifier for Diabetic Retinopathy Detection. Sensors.

[29] Liu J., Xu H., Chen Q., Zhang T., Sheng W., Huang Q., Song J., Huang D., Lan L., Li Y. (2019). Prediction of hematoma expansion in spontaneous intracerebral hemorrhage using support vector machine. EBioMedicine.

[30] Cheng X., Zhang W., Wu M.L., Jiang N., Ni Guo Z., Leng X., Song J.N., Jin H., Sun X., Zhang F. (2021). A prediction of hematoma expansion in hemorrhagic patients using a novel dual-modal machine learning strategy. Physiol. Meas..

[31] Song Z., Guo D., Tang Z., Liu H., Li X., Luo S., Yao X., Song W., Song J., Zhou Z. (2021). Noncontrast Computed Tomography-Based Radiomics Analysis in Discriminating Early Hematoma Expansion after Spontaneous Intracerebral Hemorrhage. Korean J. Radiol..

[32] Teng L., Ren Q., Zhang P., Wu Z., Guo W., Ren T. (2021). Artificial Intelligence Can Effectively Predict Early Hematoma Expansion of Intracerebral Hemorrhage Analyzing Noncontrast Computed Tomography Image. Front. Aging Neurosci..

